# Explorations into Peptide Nucleic Acid Contrast Agents as Emerging Scaffolds for Breakthrough Solutions in Medical Imaging and Diagnosis

**DOI:** 10.1021/acsomega.1c03994

**Published:** 2021-10-19

**Authors:** Rüdiger M. Exner, Stephen J. Paisey, James E. Redman, Sofia I. Pascu

**Affiliations:** † Department of Chemistry, University of Bath, Claverton Down, Bath BA2 7AY, United Kingdom; ‡ Wales Research & Diagnostic Positron Emission Tomography Imaging Centre (PETIC), School of Medicine, Cardiff University, University Hospital of Wales, Heath Park, Cardiff CF14 4XN, United Kingdom; § School of Chemistry, Cardiff University, Main Building, Park Place, Cardiff CF10 3AT, United Kingdom; ∥ Centre for Sustainable and Circular Technologies, 1 South, University of Bath, Claverton Down, Bath BA2 7AY, United Kingdom; ⊥ Centre for Therapeutic Innovation, 3 West 2.03, University of Bath, Claverton Down, Bath BA2 7AY, United Kingdom

## Abstract

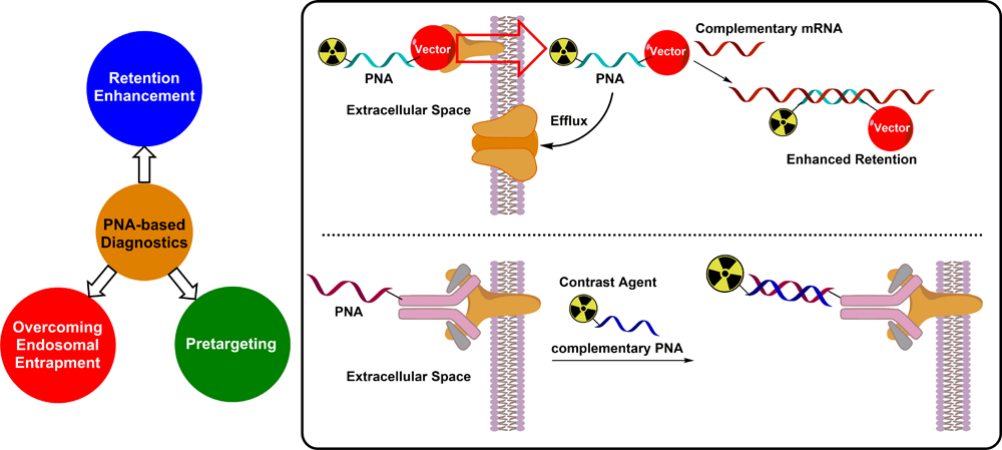

Peptide nucleic acids (PNAs, nucleic acid analogues with a peptide backbone rather than a phosphoribosyl backbone) have emerged as promising chemical agents in antigene or antisense therapeutics, as splicing modulators or in gene editing. Their main benefits, compared to DNA or RNA agents, are their biochemical stability and the lack of negative charges throughout the backbone, leading to negligible electrostatic interaction with the strand with which they are hybridizing. As a result, hybridization of PNA strands with DNA or RNA strands leads to higher binding energies and melting temperatures. A lack of natural transporters, however, necessitates the formation of PNA-containing chimeras or the formulation of nanoparticular cell delivery methods. Here, we set out to explore the progress made in using imaging agents based on PNAs in diagnostic applications and highlight selected developments and challenges.

## Introduction

**Scheme 1:**
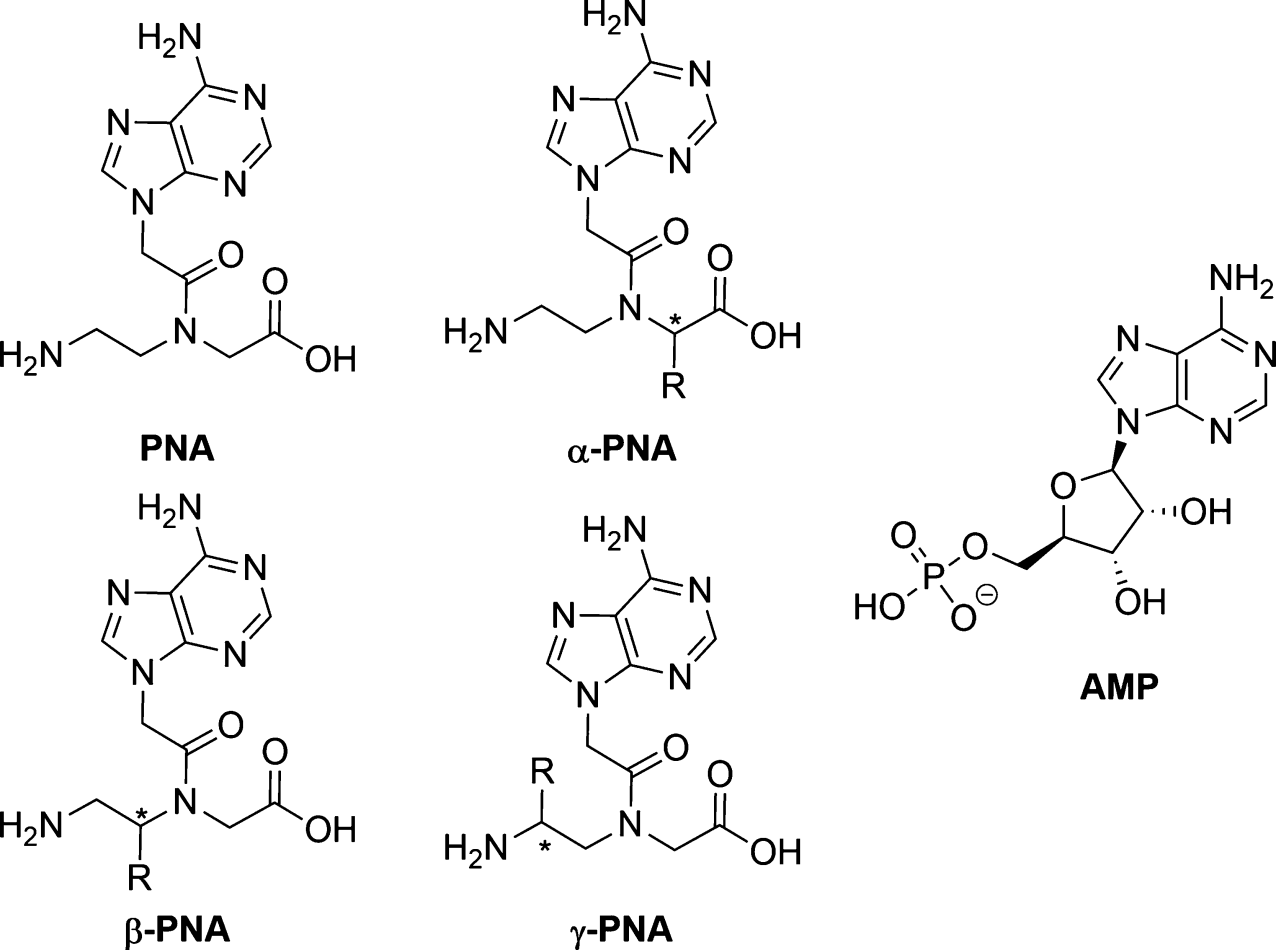
Structures of Common PNA Backbones (Depicted Here with Adenine as the Nucleobase and Adenosine Monophosphate for Comparison)

Peptide nucleic acids (PNAs), a class of xeno-nucleic acids (XNAs), have received significant amounts of academic interest over recent years.^1,2^ In contrast to their natural analogues, the nucleic acids, they do not contain a phosphoribosyl backbone but an artificial amino acid backbone, typically based on *N*-(2-aminoethyl)glycine (AEG). As a result, PNA strands can be prepared using well-established solid-phase peptide synthesis (SPPS). In addition, variations of the original AEG backbone have been reported, which modify properties of the PNA strand. Scheme 1 shows an overview of some common structural motifs, with the nucleobase adenine, as an example.

Gupta et al. as well as Sharma et al. reviewed various derivatives and selected aspects, such that these will not be discussed further hereby.^1,2^ Due to the fact that PNA strands do not seem to occur in nature, no efficient pathway to break them down has evolved in biological systems. As a result, they are easier to handle and possess in vivo kinetic stability higher than that of DNA- or RNA-based agents.^3^ The lack of negative charges from the AEG backbone of a PNA strand results in little to no electrostatic repulsion with complementary DNA or RNA single strands. As a result, PNA/DNA or PNA/RNA double strands display hybridization energies and melting temperatures significantly higher than those of DNA or RNA double strands. In addition, the lack of negative charges allows for a more efficient formation of triple helices (triplex formation), which are involved in DNA recombination and gene editing.^4^

**Figure 1:**
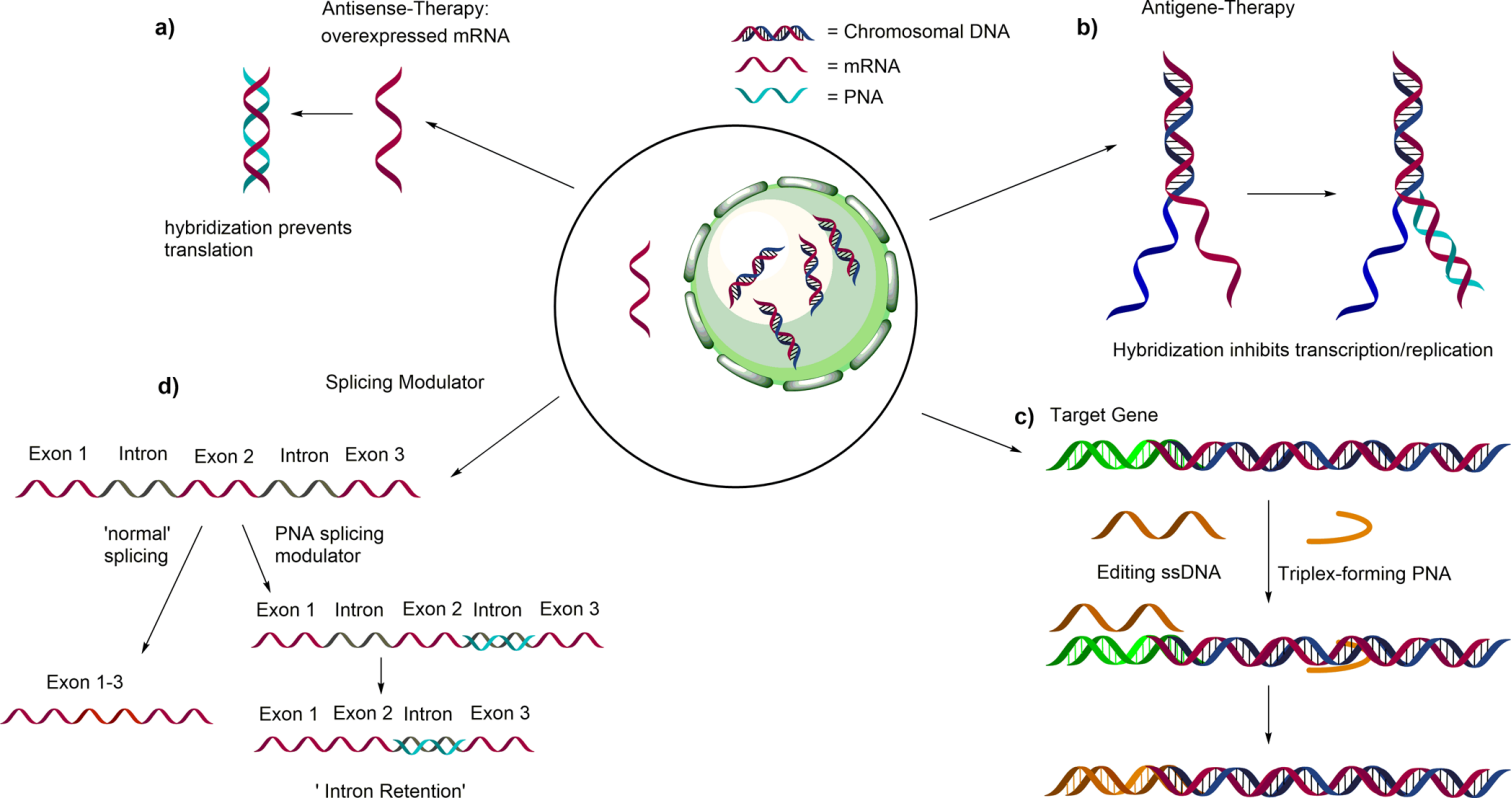
Schematic overview of PNA-based therapeutic approaches. (a) Antisense approach: binding to mRNA reduces/stops translation into functional protein. (b) Antigene therapy: transcription of DNA to mRNA is inhibited. (c) Gene editing: mediated through the use of DNA_2_PNA triplex formation in the presence of a suitable ssDNA molecule. (d) Intron retention: PNA binds to splice sites and leads to intron retention, potentially reducing the amount of functional protein.

There have been numerous in vitro studies, along with some promising preclinical studies in animal models, to investigate their potential in the treatment and management of various diseases. PNA-based agents may be used as antisense or antigene agents or as splicing modulators (Figure 1).^5,6^ There are a few reports of their use in gene-editing applications, although the exact mechanism of this has not been elucidated yet.^4^ As a result of their broad applicability, they have been evaluated as agents for the treatment of various cancers, hereditary diseases, such as Huntington’s disease, and as novel antivirals.^7−11^

Apart from exploring their therapeutic use, some research groups have also demonstrated and reported their potential as diagnostic markers, essentially using PNAs as retention enhancers for a conjugated imaging motif, thus enabling PNAs to act as theranostics. Mammalian cell lines lack transport mechanisms for them and uptake of native PNAs in eukaryotic cells is low.^12^ As such, PNAs without additional vectors or delivery mechanisms show little to no pharmacological effect.^12^ In addition, they typically distribute swiftly and are excreted quickly compared to larger biomolecules.^13,14^ To counteract these features, a delivery mechanism must be implemented. This can be realized by formation of a PNA–vector chimera or by developing appropriate nanoparticle formulations.

Here, we present an overview of the use of PNAs as synthetic building blocks for imaging agents, highlighting the state of the art and challenges involved in probe design and testing and underlining the current progress being made in both basic research and clinical settings. The term contrast agent in this context refers to compounds which may be used in any imaging modality.

## PNA-Based Chimera-Type Imaging Agents

**Figure 2:**
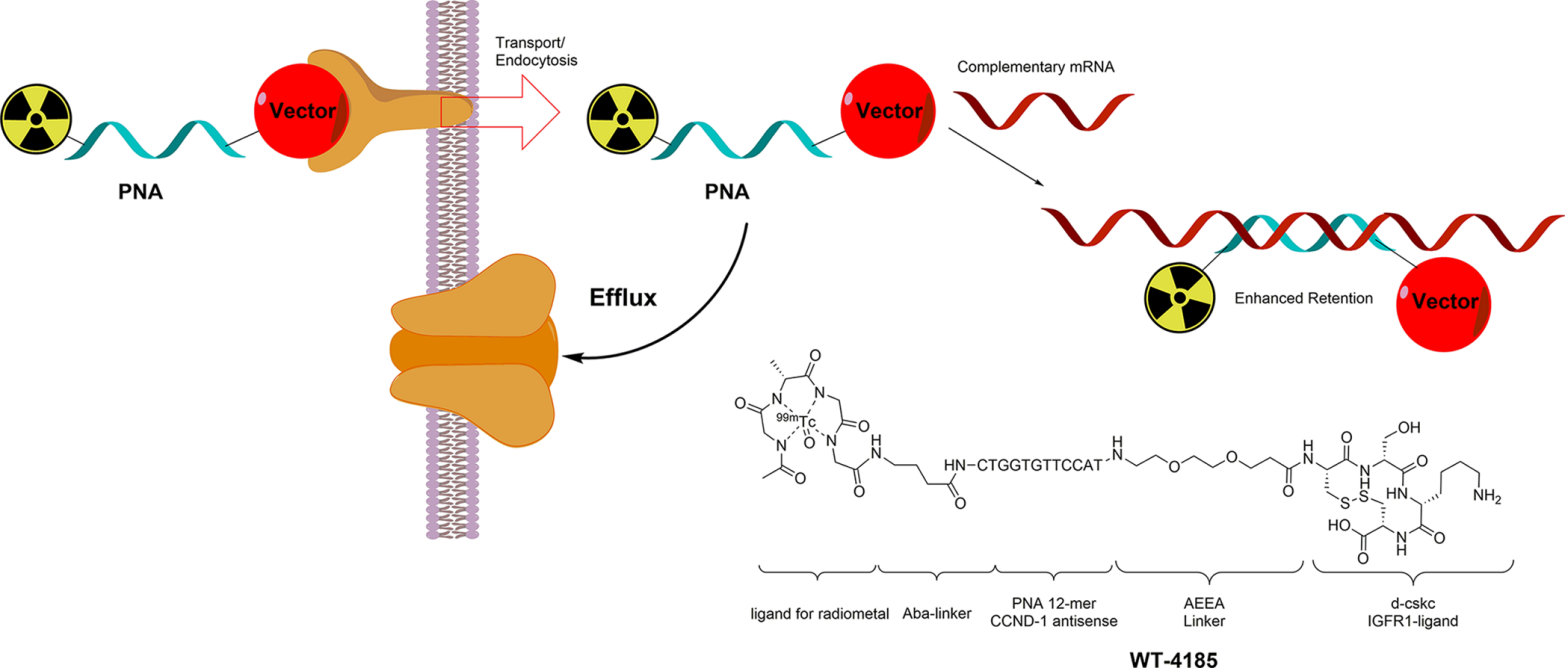
Schematic depiction of PNA-promoted retention enhancement by binding to (upregulated) mRNA. Structure of WT-4185 depicted as an example. Different functional areas of WT-4185 are indicated. The vector binds to a membrane receptor and facilitates uptake. PNA sequence binds to complementary mRNA. The ligand for a radiometal allows for nuclear imaging.

As mentioned above, a delivery mechanism is necessary to facilitate uptake of PNAs into the cytoplasm (or the nucleus, respectively). One way to achieve this is the formulation of a chimera-type imaging agent. A PNA chimera comprises the actual PNA fragment, a functionality which facilitates molecular imaging (e.g., a radioactive isotope or a fluorophore), and a vector for targeting transporters or receptors on the cell surface, typically in the form of a peptide (fragment). Compounds of this nature cannot be classified as small molecules, as their masses typically range from 4 to 6 kDa. As such, characterization is primarily done by HPLC/MS and binding assays on complementary immobilized DNA, as well as by variable-temperature UV/vis spectroscopy.^12,14^ Such chimeras are hypothesized to improve signal-to-background in imaging applications by acting as retention enhancers (Figure 2). In a typical example, such as *WT-4185* (depicted in Figure 2), a vector may target an overexpressed receptor, giving an initial selectivity for the targeted cell line.^17^ After transport into the cytoplasm, the PNA moiety would bind to the transcribed mRNA of an upregulated or mutated gene. This binding appears to enhance the retention, relative to cells that do not express the targeted gene.^17^ As an alternative to mRNA, overexpressed miRNAs may also be targeted.^15,16^

The particular benefit of developing chimera-type imaging agents then is the high versatility and modularity, which can be achieved with such agents. The same PNA fragment can, at least theoretically, be combined with any vector and any imaging modality. Similarly, the PNA fragment in such a design can be changed as needed, without affecting the overall methodology used to produce the resulting chimera. In addition, such constructs have been shown, despite their size, to exhibit rapid biodistribution.^13,14^Table 1 lists a selection of PNA–construct imaging agents and indicates the different functional moieties present within these constructs. The well-known Ac-Gly-ala-Gly-Gly ligand is also incorporated as a design element for labeling with ^99m^Tc to enable single photon emission computed tomography (SPECT).^17^ Small linkers were introduced between the different functionalities to minimize interactions between them.

**Table 1:** Selected PNA Constructs for Imaging Purposes^a^

probe	imaging modality	PNA fragment	vector	target RNA	vector target	ref
WT-4185	SPECT (^99m^Tc)	CTGGTGTTCCAT	d(cys-ser-lys-cys)	CCND1	IGF1R	(17,18)
WT-4348	PET (^64^Cu)	CTGGTGTTCCAT	d(cys-ser-lys-cys)	CCND1	IGF1R	(17,18)
WT-4433	FI (Fluorescein)	CTGGTGTTCCAT	d(cys-ser-lys-cys)	CCND1	IGF1R	(17,18)
WT-4219	SPECT (^99m^Tc)	GCATCGTCGCGG	d(cys-ser-lys-cys)	MYC	IGF1R	(18)
WT-4351	PET (^64^Cu)	GCCAACAGCTCC	d(cys-ser-lys-cys)	KRAS	IGF1R	(18)
[^64^Cu]Cu-DOTA-Anti-miR-146a	PET (^64^Cu)	AACCCATGGAATTCAGTT	Arg_8_	mi-RNA146a	- (CPP)	(15)
[^177^Lu]Lu-HP18	SPECT (^177^Lu)	ATCATCAACACCAGG	see main text	see main text	HER2	(19)

a PNA sequences given in N → C direction; peptide sequences given in N → C direction. CPP = cell-penetrating peptide; miRNA = micro-RNA. The following naming conventions are used: nucleobases are abbreviated as capital letters (A(denine), C(ytosine), G(uanine), T(hymine)). Amino acids are written in three letter codes, with the first letter capitalized for l-amino acids or in lower case for d-amino acids. The lowercase prefix d refers to cyclization via disulfide bonds, whereas the lowercase prefix c refers to cyclization via amide bond formation.

**Figure 3:**
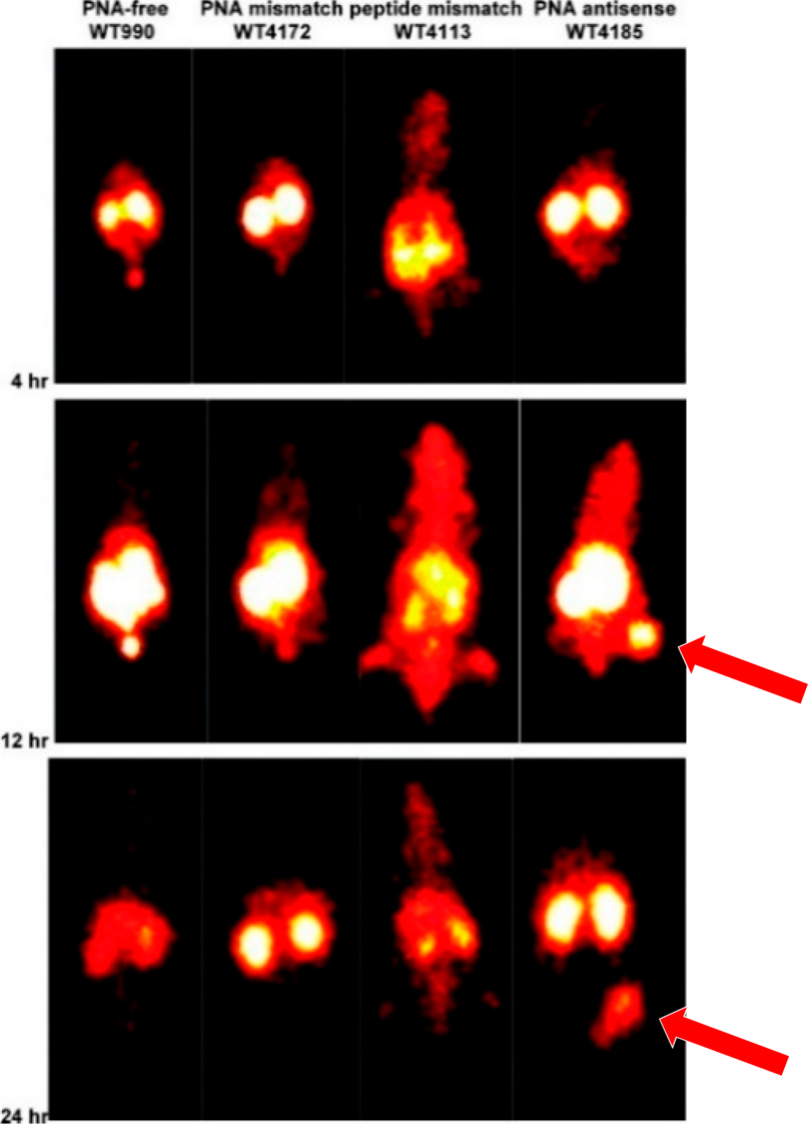
Scintigraphy (γ) obtained with various ^99m^Tc-labeled (PNA) constructs, indicating that antisense PNA-based chimeras (WT4185) lead to retention in cells (over)expressing complementary mRNA. Xenograft site is indicated by the arrow. This figure is modified here, by addition of arrows, with permission from ref (17). Copyright 2004 Society of Nuclear Medicine & Molecular Imaging.

Typical choices of linkers included in the constructs highlighted in Table 1 are aminobutyric acid (Aba) and [2-(2-(amino)ethoxy)ethoxy]acetic acid (AEEA).^15,18^ To assess the effect of a PNA-based probe, experiments with a mismatched analogue and ideally a PNA-free analogue are necessary, and a number of further experiments are possible to demonstrate that binding to complementary mRNA, rather than other factors, improves retention in the targeted tissue. For instance, Tian et al. developed *WT-4433*, an analogue of *TW-4185*, in which the ligand moiety is replaced by fluorescein. The fluorescent probes were used in cell-imaging assays and indicated promising results when compared to the mismatched derivative WT-4361.^17,18^ A xenograft study in mice showed that the PNA chimeras accumulated in the tumor and could still be detected after 24 h. Control experiments featuring a PNA-free peptide–ligand conjugate (*WT-990*) showed little to no signal at the time points chosen in this study (Figure 3). Similarly, a PNA mismatch (*WT-4172*) and a different cyclized peptide (*WT-4113*, peptide sequence d(cys-ala-ala-cys)) showed reduced tumor-to-background ratios.^17^ These results suggest that the PNA may, in fact, lead to increased retention in cells. Furthermore, the results obtained with PNA mismatch probes indicate that binding to mRNA indeed plays a role in the enhanced retention and in vivo distribution. Western blots of the CCD1 protein showed reduced quantities of protein in mice treated with the antisense PNA, compared to the mismatched or PNA-free analogues, further indicating that binding between PNA and mRNA occurred in vivo.^17^ Noticeably, the probes featuring the ^99m^Tc ligand and d(cys-ser-lys-cys) show significant kidney uptake. This is likely a feature of both functionalities, rather than an inherent feature of PNA chimeras. Other ^99m^Tc tetrapeptide ligands are well-known for facilitating urinary excretion.^20^ Similarly, expression of IGF1R in mice kidneys may be high, as even a PNA-free peptide–radioligand combination shows considerable uptake, while the peptide mismatch shows little specific uptake. Other authors working with biomolecular IGF1R imaging agents, such as affibodies, likewise observed considerable kidney uptake.^21^

Therefore, a more thorough use of bioinformatics and mRNA expression data, not only for the development of the antisense PNA but also in the choice of vectors, may be important for future progress. Chimeras featuring other ligands show lower renal and instead increased hepatic uptake.^14,18^

This indicates that the distribution and clearance behavior can likely be optimized either by changing vectors or linkers or by adjusting molecular charge and solubility. A central challenge in the design and optimization of chimeras for diagnostic purposes is the distribution on the cellular level, specifically in terms of cytosol release. Mishra et al. developed a multimodal imaging agent, comprising the d-Tat_57–49_ fragment for cell targeting, antisense PNA to DsRed2, DOTA for ligation of gadolinium(III) for magnetic resonance imaging (MRI), and fluorescein isothiocyanate (FITC) for fluorescence imaging (FI).^14^ The comparison of the antisense PNA probe to a mismatch derivative in vitro showed no significant difference in fluorescence intensity after 18 h of incubation, likely due to endosomal entrapment. As noted by other authors, entrapment in endosomes is a commonly observed issue in the delivery of these agents.^3,14^ Many cell-penetrating peptides (CPPs) will not be able to effectively translocate from endosomes or lysosomes into the cytosol.^22^ Shiraishi et al. investigated the influence of conjugates of a PNA molecule and various CPPs in vitro, using a luciferase assay in which an aberrant splicing site was targeted, such that binding of PNA led to the translation of functional luciferase protein.^23^ Their results indicated not only that the efficiency of the treatment was highly dependent on the exact CPP used but also that treatment of cells with Ca^2+^ (from CaCl_2_) improved delivery significantly.^23^

Recently, it was also demonstrated that this may be addressed by developing cyclic CPPs of the general formulas dCys-CPP-Cys, cCPP, or CPP-hemagglutinin conjugates.^24,25^ While most of these studies showed increased cytosol release in vitro, such delivery mechanisms were also suggested to have certain drawbacks such as a lack of the desired selectivity for the targeted cells, instead relying exclusively on the PNA-based retention enhancement, potentially limiting the achievable contrast. As such, development of release mechanisms into the cytoplasm is of utmost importance to develop effective PNA-based therapeutic or imaging agents, giving rise to further challenges in their development. There are numerous unexplored avenues for the development of PNA-based chimeras. Only a small number of vectors and imaging agents have been explored so far in terms of their diagnostic capability in disease models in vivo.

Optimization of clearance and biodistribution behavior in vivo is of great importance to make such constructs applicable to imaging of cancers or following in real time disease biomarkers. Development of reliable delivery methods into the cytoplasm is necessary to realize the full potential of this class of probes. Finding general synthetic and imaging strategies to meet these challenges would allow for the development of new multimodal imaging agents, agents for nuclear therapy or gadolinium or boron neutron capture therapy, which could capitalize on the enhanced retention successfully demonstrated by some authors.

## Pretargeting Studies Using PNA-Based Imaging Probes

**Figure 4:**
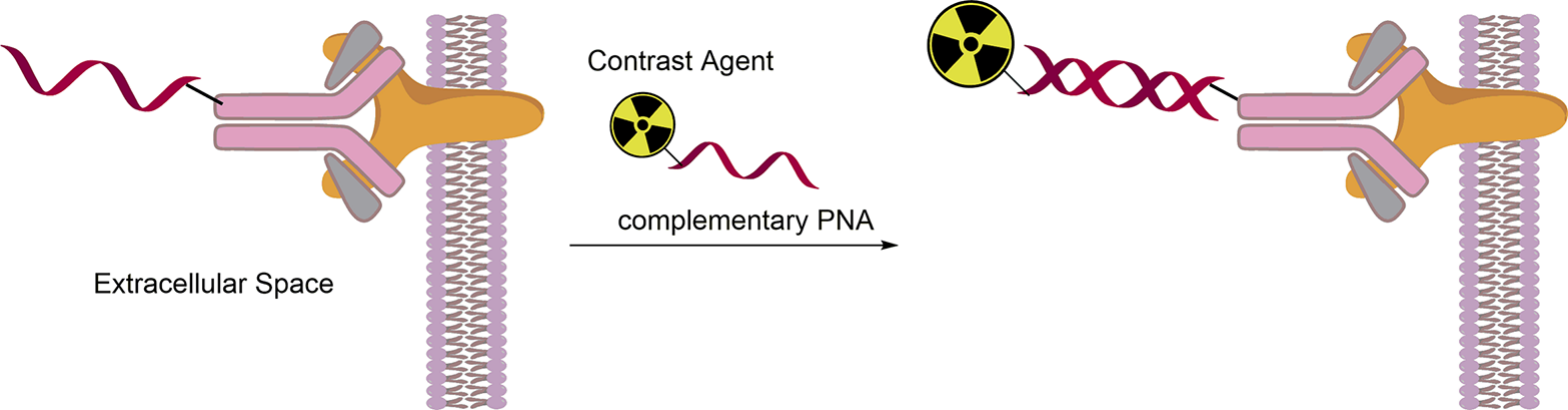
Schematic depiction of PNA-promoted pretargeting. In a pretargeting approach, PNAs recognize complementary PNA strands in the extracellular space.

As an alternative to the use of PNA chimeras, PNAs may also be used as motifs for supramolecular recognition in pretargeting approaches.^19,26,27^ A schematic representation can be found in Figure 4. Pretargeting approaches typically rely on either bioorthogonal click reactions in vitro or in vivo, such as the inverse electron-demand Diels–Alder (IEDDA) reaction between *trans*-cyclooctene and tetrazine,^28^ or on the supramolecular recognition of a host and a guest, such as the interaction between adamantane and cyclodextrin.^29^ In contrast to other methods, pretargeting approaches rely on presentation of the recognition motif on the cell surface. As such, successful approaches usually rely on large biomolecules, such as monoclonal antibodies or affibodies, to avoid the quick internalization typically observed for small molecules and peptides. This alleviates the need for developing endosomal escape strategies and allows one to focus on other challenges, like the overall biodistribution and excretion of unbound probes. Several authors already successfully demonstrated the use of such a strategy involving PNAs.^19,30^

To the best of our knowledge, the first report of PNA-based pretargeting was published in 1997 and demonstrated its potential in both infection and tumor models.^31^ More recent work performed by Leonidova et al. demonstrated the use of a cetuximab–PNA conjugate for targeting of cancers overexpressing the endothelial growth factor receptor (EGFR).^30^ Similarly, the use of a trastuzumab–affibody fusion protein, synthesized using UV click chemistry and featuring a PNA strand conjugated to the affibody, has been explored.^27^

**Figure 5:**
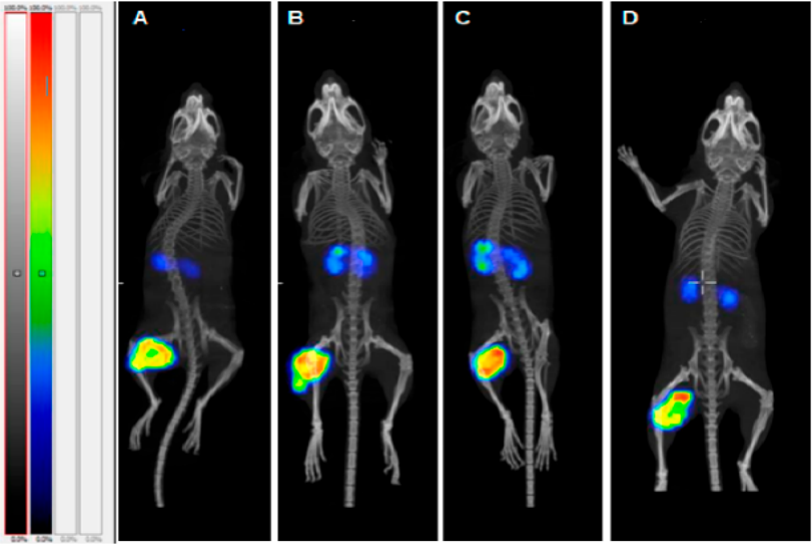
SPECT/CT imaging via pretargeting of mice bearing HER2 positive SKOV3 ovarian cancer xenografts. Mice were pretreated with a HER2-affibody–PNA conjugate (Z_HER2:342_-SR-HP15 in A–C; Z_HER2:342_-SR-HP1 in D). After 16 h, lutetium-177-labeled PNA–DOTA constructs were injected (A = [^177^Lu]-Lu-HP16 (9 nucleobases), B = [^177^Lu]-Lu-HP17 (12 nucleobases), C = [^177^Lu]-Lu-HP18 (15 nucleobases), D = [^177^Lu]-Lu-HP2). After a further 4 h, imaging was performed. Reproduced with permission from ref (19). Copyright 2021 MDPI.^19^

Tano et al. synthesized a conjugate of a PNA molecule and a HER2-targeting affibody, expanding the investigated biomolecules further.^19^ After allowing for initial biodistribution of the affibody conjugate, lutetium-177-labeled PNA constructs of various length were injected. SPECT/CT images obtained as part of this study are depicted in Figure 5.

Noticeably, the shorter PNA constructs produced higher tumor-to-kidney ratios in xenograft studies, presumably due to favorable pharmacokinetics.^19^ As with some other pretargeting approaches, considerable washout was observed, potentially limiting the therapeutic applicability. Nevertheless, for diagnostic purposes, such a strategy seems promising. Other biomolecules or fragments are, to the best of our knowledge, hitherto unexplored for PNA-based pretargeting. However, the use of single domain antibodies (nanobodies), diabodies, or isolated antigen-binding fragments (Fab) could be of interest to generate conjugates with better biodistribution and excretion dynamics.

Similarly, the rapid clearance of unbound radiolabeled PNA constructs in vivo is desirable. Several authors have investigated the distribution behavior of these constructs in the absence of biomolecules decorated with the complementary PNA fragment. The available results suggest that shorter or PEGylated derivatives are more rapidly cleared, with clearance predominantly occurring via the urinary tract.^19,26,30^ Alternative (α,β,γ-functionalized) PNA derivatives with improved solubility and/or reduced self-aggregation behavior may further improve excretion of an unbound PNA-based probe.

## Challenges and Opportunities in PNA Probe Design: Toward Nano-Theranostics

Although PNA-based chimeras are relatively straightforward to synthesize, thanks to progress in technologies such as microwave-based peptide and nucleic acid synthesizers and the highly modular aspects in the nature of building blocks relevant to the molecular design, there are aforementioned challenges regarding the delivery and subsequent cytosol distribution and biodistribution, which thus far slowed down their development for disease diagnostic purposes. In PNA constructs capable of avoiding or escaping endosomal entrapment post-cellular delivery, the binding to (overexpressed) RNA must slow down the efflux sufficiently relative to unbound PNA to contribute to contrast.

Overall, the PNA constructs reported to date typically showed little to no toxicity, both in vitro and in vivo, and are straightforward to functionalize and adapt to specific targets. Despite some promising advances, no PNA chimera has made the transition into a clinical trial yet. Effective translocation across the blood–brain barrier is a problem observed with many drug molecules and has only been demonstrated for a few of the PNA-based probes developed so far.^32−34^

The considerable renal or hepatic retention would likely render most PNA chimeras unsuitable for imaging applications in the lower abdomen if similar distribution patterns were found in humans. However, this may be addressed by choosing more suitable vectors and adjusting linkers and/or overall charge of the molecule. The development of PNA constructs for pretargeting applications may be a straightforward way to address this issue. Similarly, to other pretargeting approaches, PNA-based constructs have great potential for further development and clinical translation, due to the specificity of their interaction with a complementary PNA strand.

An alternative that may be able to address some of these problems may be the formulation of nanoparticulate PNA delivery systems or PNA nanomedicines. Synthetic developments in this area would rely heavily on the advances that have already been made in delivering RNA into the cytosol, such as in mRNA vaccines, and in designing cancer-targeting nanoparticles.^35^

Many nanoparticles are known to accumulate in cancerous tissues, which is typically explained through the debated enhanced retention and permeation hypothesis.^36^ While endosomal entrapment is usually not a problem that is commonly observed with nanoparticles, a PNA-based agent which is encapsulated would still have to be released into the cytosol to take effect. One option to achieve this is the use of lipid nanoparticles. A limitation of nanoparticle-based approaches may be that they typically show slow biodistribution, as they move through the lymphatic system. Due to this, and the significant amount of time needed for washout of unbound probe, some long-lived nonstandard radioisotopes such as ^89^Zr or ^52^Mn may be of interest for nuclear imaging applications. Alternatively, other imaging modes could be considered, such as photoacoustic imaging, fluorescence imaging, or MRI, although the lower sensitivity requires larger quantities.

Apart from targeting mRNAs, there are opportunities to target the various forms of noncoding RNAs, such as small interfering (si)RNAs or miRNAs, as demonstrated by some authors,^15,16,37^ for diagnostic purposes. It is likely that there is a great number of different siRNAs and miRNAs yet to be discovered.

Regarding optical imaging approaches capable of delivering information on PNA probes’ behavior with high resolution at the in vitro level, several modalities have been successfully demonstrated, such as the delivery of PNA/DNA binary Förster resonance energy transfer probes for visualization of splicing patterns, mRNA expression levels, or visualizing siRNA delivery into cells.^38−40^ For example, using a fluorophore/quencher system, Shigeto et al. were able to differentiate different lung cancer cell lines by fluorescence microscopy.^41^ Using PNA-based imaging agents in this context may aid in the development of nucleic-acid-based therapies, by visualizing successful delivery of active agents in preclinical development.

Overall, PNA-based imaging agents have a number of potential diagnostic imaging applications, additional to therapeutic applications, both in vitro and in vivo. Their high stability renders these probes rather straightforward to handle in drug delivery approaches, unlike RNA molecules which are readily degraded by the virtually ubiquitous RNases, and their high hybridization energies with complementary PNA, RNA, or DNA strands leads to a plethora of therapeutic and diagnostic opportunities.
